# The *MNN2* Gene Knockout Modulates the Antifungal Resistance of Biofilms of *Candida glabrata*

**DOI:** 10.3390/biom8040130

**Published:** 2018-10-30

**Authors:** Célia F. Rodrigues, Diana Vilas Boas, Ken Haynes, Mariana Henriques

**Affiliations:** 1 CEB, Centre of Biological Engineering, LIBRO—Laboratório de Investigação em Biofilmes Rosário Oliveira, University of Minho, 4710-057 Braga, Portugal; dianavilasboas@ceb.uminho.pt (D.V.B.); mcrh@deb.uminho.pt (M.H.); 2 Biosciences, College of Life and Environmental Sciences, University of Exeter, Exeter EX4 4QD, Devon, UK; k.haynes@exeter.ac.uk

**Keywords:** *Candida glabrata*, *MNN2* gene, antifungal, resistance, biofilm matrix

## Abstract

*Candida glabrata* biofilms are recognized to have high resistance to antifungals. In order to understand the effect of mannans in the resistance profile of *C. glabrata* mature biofilms, *C. glabrata* Δ*mnn2* was evaluated. Biofilm cell walls were analysed by confocal laser scanning microscopy (CLSM) and their susceptibility was assessed for fluconazole, amphotericin B, caspofungin, and micafungin. Crystal violet and Alcian Blue methods were performed to quantify the biomass and the mannans concentration in the biofilm cells and matrices, respectively. The concentration of β-1,3 glucans was also measured. No visible differences were detected among cell walls of the strains, but the mutant had a high biomass reduction, after a drug stress. When compared with the reference strain, it was detected a decrease in the susceptibility of the biofilm cells and an increase of β-1,3 glucans in the *C. glabrata* Δ*mnn2*. The deletion of the *MNN2* gene in *C. glabrata* induces biofilm matrix and cell wall variabilities that increase the resistance to the antifungal drug treatments. The rise of β-1,3 glucans appears to have a role in this effect.

## 1. Introduction

In opposition to *Candida albicans* and other *Candida* species, *Candida glabrata* grows only as blastoconidia, and has a haploid genome [1]. Despite being a part of the natural human microflora, under circumstances where the host immune system becomes severely compromised (e.g., chemotherapy, traumas), *C. glabrata* can invade epithelial cells, disseminating via the bloodstream, causing systemic diseases [1,2]. In fact, fungal diseases associated with non-*Candida albicans Candida* (NCAC) species have been globally increasing [3,4]. The frequency rates of candidemia attributed to *C. glabrata* are about 15% of all *Candida* species-related systemic bloodstream infections [5,6], which is particularly relevant since, compared with other *Candida* species infections, the mortality rate associated with *C. glabrata* is the highest (30%) [1,7]. As a matter of fact, the biofilm formation is a very important virulence factor for *C. glabrata*, allowing the yeasts to adhere to biotic and abiotic surfaces, developing into organized communities that are extremely refractory to antifungal treatment and environmental conditions [8].

The yeast cell wall is a dynamic structural organelle crucial for protection against hostile environments, maintaining cell shape, assisting adherence to host surfaces, and having a fundamental role for fungus-host interactions and immune recognition [2,9]. Per se, it is very likely to play an important part in mediating several interactions and hence virulence [9]. The cell wall is comprised of an internal central core of β-1,3 glucans and β-1,6 glucans linked to chitin via β-1,4 glucans, extended throughout the entire depth of the cell wall structure [10,11]. There is also an external layer of highly glycosylated mannoproteins [10,12], which play a major role in host recognition, adhesion, cell wall integrity, and include up to 40% of the cell wall dry weight [13,14]. These proteins are adorned with both N- and O-linked sugars, mainly mannans, and can end in the accumulation of up to 200 mannose units [9,15], attached via a phosphodiester linkage (phosphomannan) [16]. The number of mannans units and their molecular weights highly fluctuate between species [17], which have important consequences for host-fungus interactions [2,9]. Structural studies point out that *C. glabrata* mannan is more closely related to that of *Saccharomyces cerevisiae* than to *C. albicans* [18]. Even though, the core of the biosynthetic machinery appears to be relatively well conserved [9]. The N-linked protein glycosylation occurs in two stages. Concisely, in the first, proteins transverse though the endoplasmic reticulum, resulting in the mature N-mannan core, Man_8_GlcNAc_2_ [19]; in the second, a α-1,6-backbone is attached to a Och_1_ core and Mnn_9_ and added with side chains consisting of α-1,2-, and α-1,3-mannose residues, with the α-1,2-mannose extending out from the backbone [20]. It is recognised that the deletion of Sc*MNN2* inhibits the accumulation of α-1,2-mannose onto the mannan backbone, hence stopping the elaboration of N-mannan outer chains [21]. In *C. albicans*, this greatly conditions the growth, cell morphology, and immune recognition [20]. Several mannosyltransferases involved in mannan biosynthesis in *S. cerevisiae*, have been identified, and many of these enzymes are conserved in *C. albicans* and other pathogenic fungi. Remarkably, many of these fungal mannosyltransferases are absent from human cells, and thus its study shows prospective in the development of novel antifungals and vaccines. Yet, this subject is more advanced in *S. cerevisiae* and *C. albicans*, but little is known regarding the role in the pathogenesis of *C. glabrata*, especially in biofilms.

This work aims to evaluate and to understand the responsibility of mannans on the biofilm resistance of antifungals in *C. glabrata*, through the effect of a *MNN2* knock out (KO)–a putative component of the N-linked glycosylation machinery on matured biofilm cells, on their cell wall and after different drug stresses.

## 2. Material and Methods

### 2.1. Organisms

Three strains of *C. glabrata* were used in the course of this study. One reference strain from the American Type Culture Collection (*C. glabrata* ATCC2001) and two strains kindly provided by Dr. Ken Haynes from the University of Exeter (United Kingdom) [9]: the *C. glabrata* mutant with a *MNN2* gene disruption (*C. glabrata* Δ*mnn2*) and its parent strain (*C. glabrata* ΔHT6) (see Table S1 of Supplementary Material). The effect of the revertant strain was already stated and reported previously by Dr. Ken Haynes lab [9].

The identity of all isolates was confirmed using CHROMagar Candida (CHROMagar, Paris, France) and by PCR-based sequencing [22]. The PCR products were sequenced using the ABI-PRISM Big Dye terminator cycle sequencing kit (Perkin Elmer, Applied Biosystems, Warrington, UK).

### 2.2. Growth Conditions

For each experiment, *C. glabrata* ATCC2001 and *C. glabrata* ΔHT6 strains were subcultured on Sabouraud dextrose agar (SDA) (Merck, Darmstadt, Germany) and the mutant *C. glabrata* Δ*mnn2* was cultured on SD-his, as indicated by West et al. [9], during 24 h at 37 °C. Cells were then inoculated in Sabouraud dextrose broth (SDB, Merck) and incubated for 18 h at 37 °C under agitation at 120 rpm. After incubation, the cells were harvested by centrifugation at 3000 g for 10 min at 4 °C and washed twice with phosphate buffered saline (PBS 0.1M, pH = 7.5). Pellets were then suspended in RPMI-1640 (Sigma-Aldrich, Roswell Park, St. Louis, MO, USA) and the cellular density was adjusted to 1 × 10^5^ cells/mL, using a Neubauer counting chamber.

### 2.3. Antifungal Drugs

Fluconazole (Flu), caspofungin (Csf) and micafungin (Mcf) were kindly provided by Pfizer, S.A. (New York, NY, USA), MSD (Kenilworth, NJ, USA) and Astellas (Tokyo, Japan) respectively, in its pure compound. Amphotericin B (AmB) was purchased in Sigma. Aliquots of 5000 mg/L were prepared using dimethyl-sulfoxide (DMSO). The final concentrations used were prepared with RPMI-1640.

### 2.4. Biofilm Matrix Structure: Confocal Laser Scanning Microscopy

Biofilms were formed in 24-well polystyrene microtiter plates (Orange Scientific, Braine-l’Alleud, Belgium), on plastic highly adhesive round tissue culture coverslips (13 mm diameter, Sarstedt, Germany). For this, 1000 µL of yeast cell suspension (1 × 10^5^ cells/mL in RPMI-1640) were added to each well and the biofilms performed as described previously [23]. After 24 h, 500 µL of RPMI medium was removed and an equal volume of fresh RPMI with or without the antifungal agents was added.

The staining and confocal laser scanning microscopy (CLSM) were performed similarly to a method described previously [24]. Biofilms were fixed in 4% (*w*/*v*) of paraformaldehyde (Sigma-Aldrich) followed by 50% (*v*/*v*) ethanol for 10 min at room temperature and allowed to air dry. Then, to stain the α-mannopyranosyl residues of the glycosylated mannoproteins (that is, mannans) of the cells, they were overlayed with 25 μg/mL Concanavalin A-Alexa Fluor 488 conjugate (ThermoFisher Scientific, Bartlesville, OK, USA) in the dark for 10 min at room temperature [10,12,25]. For cell nucleus detection, the cells were stained with 100 mg/L of 4′,6-diamidino-2-phenylindole (DAPI, ThermoFisher Scientific) for 10 min at room temperature.

After this, the preparation was washed with water and allowed to air dry. Finally, the sample was immediately observed using the CLSM (Olympus BX61, Model FluoView 1000, Tokyo, Japan). Images were acquired with the program FV10-ASW 4.2 (Olympus) using a magnification of ×100.

### 2.5. Biofilm Antifungal Susceptibility Tests

The antifungal susceptibility tests were performed using the microdilution method, in accordance to the European Committee on Antimicrobial Susceptibility Testing guidelines [26,27].

The Minimum Biofilm Eradication Concentration (MBECs), that were previously determined for *C. glabrata* ATCC2001 for all drugs (Flu, AmB, Csf, and Mcf) [27,28], was used to evaluate the biofilm susceptibility of *C. glabrata* Δ*mnn2* and the parent strain, *C. glabrata* ΔHT6. For that, standardized cell suspensions (200 μL) were placed into selected wells of 96-wells polystyrene microtiter plates (Orange Scientific). RPMI-1640 was used without cells, but with antifungal agent, as a negative control. As positive control, cell suspensions were tested without antifungal agent. At 24 h, 100 μL of RPMI-1640 was removed and an equal volume of fresh RPMI-1640 plus the respective antifungal concentration were added (Flu: 1250 mg/L; AmB: 4 mg/L; Csf: 3 mg/L; Mcf: 17 mg/L–2x concentrated). The plates were incubated at 37 °C for more 24 h, a total of 48 h, at 120 rpm. The number of cultivable cells on biofilms was determined by the enumeration of colony forming units (CFUs). For that, after the period of biofilm formation, all medium was aspired and the biofilms washed once with 200 μL of PBS to remove non-adherent cells. Then, biofilms were scraped from the wells and the suspensions were vigorously vortexed for 2 min to disaggregate cells from the matrix. Serial decimal dilutions in PBS were plated on SDA and incubated for 24 h at 37 °C [29]. The results were presented in percentage of biofilm cell reduction.

Total biofilm biomass was quantified by Crystal Violet (CV) staining [28]. After biofilms formation, the medium was aspirated and non-adherent cells removed by washing the biofilms with sterile ultra-pure water. Then, biofilms were fixed with 200 μL methanol, which was removed after 15 min of contact. The microtiter plates were allowed to dry at room temperature, and 200 μL of CV (1% *v*/*v*) were added to each well and incubated for 5 min. Then, wells were gently washed twice with sterile, ultra-pure water and 200 μL of acetic acid (33% *v*/*v*) were added to release and dissolve the stain. The absorbance of the obtained solution was read in a microtiter plate reader (Bio-Tek Synergy HT, Izasa, Lisbon, Portugal) at 570 nm. The results were presented as absorbance per unit area (Abs/cm^2^).

### 2.6. Effect of the Antifungals on Biofilm Cells’ Walls and on Biofilm Matrix Composition

#### 2.6.1. Matrix Extraction Method

Biofilms were formed as explained above. After 48 h, biofilms were scraped from the 24-well plates, resuspended in ultra-pure water, sonicated (Ultrasonic Processor, Cole-Parmer, IL, USA) for 30 s at 30 W, and then the suspension vortexed for 2 min. The suspension was centrifuged at 5000 g for 5 min at 4 °C and the supernatant filtered through a 0.2 μm nitrocellulose filter. The pellets were dried at 37 °C until a constant dry biofilm weight was achieved.

#### 2.6.2. Quantification of Mannans on Biofilm Cells and on Biomass—Quantitative Alcian Blue Binding Assay

Alcian Blue assay was performed according previously described by Odani et al. [30] with slight adjustments [24]. Regarding the mannans on cell membranes, biofilms of *C. glabrata* strains were grown as previously described, scraped and sonicated (Ultrasonic Processor) for 30 s at 30 W, and then the suspension vortexed for 2 min, to separate cells form the biofilm matrices. Next, the isolated cells were washed with deionized water, the *OD_600_* read and adjusted at an *OD_600_* of 0.6 in deionized water. Afterwards, the cells were washed with 0.02 N HCl (pH = 3), the pellet was suspended in 1 mL of 30 μg/mL Alcian blue dye dissolved in 0.02 N HCl (pH = 3) and incubated for 10 min at room temperature. Then, the cells were pelleted and evaluated for dye binding. To quantify dye binding and dye remaining in the supernatant after binding the stain was measured spectrophotometrically at 600 nm. To calculate the Alcian Blue Binding the following equations were used:

Amount of dye adsorbed to the cells (*T*): *T* (μg) = 61.3 × (*OD_600 ori_ − OD_600 sup_*) (*OD_600 ori_*—OD of the Alcian Blue Dye working solution; *OD_600 sup_*—OD of the supernatant);

Alcian Blue Binding (μg/*OD_600_*) = (*T* × *d*)/*OD_600 c.c._*

(OD_c.c._—the exact OD reading; d—dilution factor).

#### 2.6.3. Alcian Blue Quantitative Assay on Biofilms

To measure the relative percentage of mannans on biofilm (total biomass), the medium with planktonic cells was all removed from the well and the biofilm was washed with 0.02 N HCl (pH = 3). Next, the biofilm was scraped, suspended in 1 mL of 30 μg/mL Alcian blue dye dissolved in 0.02 N HCl (pH = 3), incubated for 10 min at room temperature and pelleted for evaluation of dye binding. To calculate relative percentage of mannans on biofilm the following equation was used:

% mannans on biofilm: [(*OD_600 ori_* − *OD_600 sup_*)/*OD_600 ori_*] × 100

(*OD_600 ori_*—OD of the Alcian Blue Dye working solution; *OD_600 sup_*—OD of the supernatant).

#### 2.6.4. Isolation of *Candida glabrata* Biofilm Cells Walls

Cell wall fractionation was performed as described by Pitarch et al. [31] with slight modifications. For that, biofilms were prepared as described above. *C. glabrata* biofilm cells were collected by centrifugation, washed two times with PBS and sonicated at 30 W for 30 s, in order to allow separation of the cells from matrix. Next, biofilm cells were separated by centrifugation at 5000 g for 5 min in a cell wall fraction (pellet) and a soluble cytoplasmic fraction (supernatant). Following this, the cell wall fraction was dissolved in 500 μL of buffer with phenylmethylsulfonyl fluoride (PMSF) (1 mM) and transferred to “lysis” tube with 500 μL of glass beads for the cells disruption of the cells in the FastPrep (MP, Biomedicals, Illkirch-Graffenstaden, France) at maximum speed for 30 s. The “lysis” tube was placed in a mixture of ice and water for 30 s. These steps were repeated seven more times. This procedure was carried out until complete cell breakage, verified beforehand by phase-contrast microscopic examinations and a posteriori by the failure of cells to grow on YPD-chloramphenicol plates. Then, the samples were transferred to microcentrifuge tubes and spin in microfuge centrifuge at 4 °C for 3 min at 2500 g. Hereafter, the isolated cell walls were washed with solutions of decreasing concentrations of NaCl (five times with each of the following ice-cold solutions: 5% NaCl, 2% NaCl, 1% NaCl, and 1 mM PMSF) to remove any extracellular or cytosolic protein contaminants that might be adhered to the cell walls through electrostatic forces. The supernatant was transferred to fresh microfuge tubes and centrifuged at 4 °C for 10 min at 10,000× *g*. The supernatants were finally transferred to fresh tubes.

#### 2.6.5. β-1,3 glucans Concentration Determination

The β-1,3 glucans concentration was determined using Glucatell kit (Cape Cod, East Falmouth, MA, USA). The values were normalized per dry weight of biofilm on biofilm matrices and per pg/mL of β-1,3 glucans on biofilm cells.

### 2.7. Statistical Analysis

The experiments were performed in triplicate and in three independent assays. The results were compared using one-way analysis of variance (ANOVA), Tukey’s post hoc multiple comparisons tests, using GraphPad Prism 7 software (GraphPad Software, San Diego, CA, USA). All tests were performed with a confidence level of 95%.

## 3. Results

**Figure 1:**
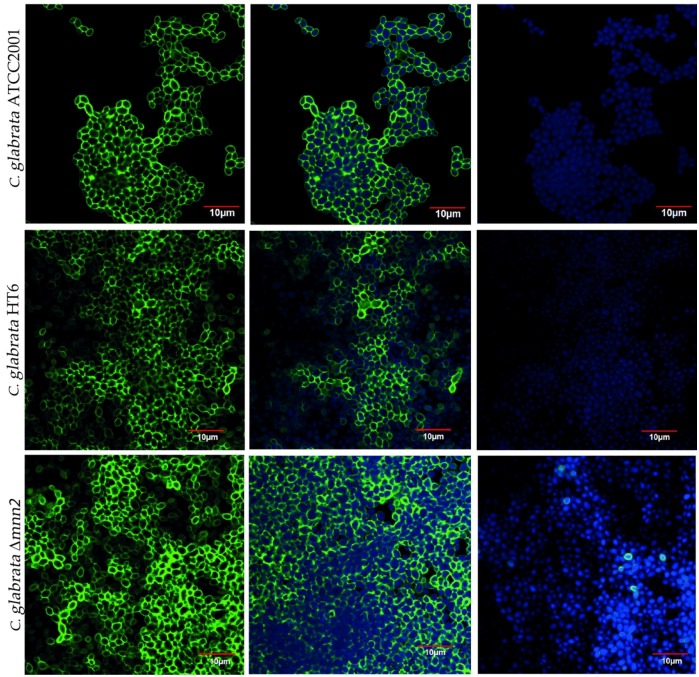
Confocal laser scanning microscopy image a 48-h-biofilm of *Candida glabrata* ATCC2001, *C. glabrata* ΔHT6 and *C. glabrata* Δ*mnn2*. The biofilm images were acquired using a confocal scanning laser microscope (Olympus BX61, Model FluoView 1000). Filters: DAPI (100 mg/L emissions filters BA 430–470) and Concanavalin A, Alexa Fluor 488 conjugate (50 mg/L emissions filters BA 505–605). Images were acquired with the program FV10-ASW 4.2 (Olympus) using a magnification of 100×. Measure bar: 10 µm.

First, with the purpose of visualizing the effect of the *MNN2* gene KO on *C. glabrata* cell walls, the biofilms were observed microscopically by CLSM (Figure 1). The images of the 48-h-biofilms of *C. glabrata* ATCC2001, *C. glabrata* Δ*mnn2*, and *C. glabrata* ΔHT6 showed that the gene deletion, apparently, had no effect on the cell wall, since there were no relevant differences among the three strains (Figure 1). All strains demonstrated to have good biofilm forming capacity, composed by yeasts in a continuous carpet [23,32,33] (Figure 1). Yet and curiously, *C. glabrata* Δ*mnn2* and *C. glabrata* ΔHT6 exhibited higher amount of multilayer structures, whereas *C. glabrata* ATCC2001 showed a less amount of yeast cells forming these structures (Figure 1).

### 3.1. Effect of the Antifungals on Biofilms

**Figure 2:**
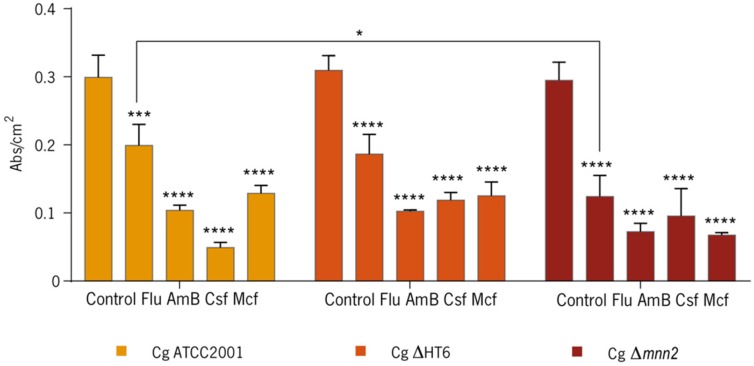
Crystal Violet in a 48-h-biofilm of *C. glabrata* ATCC2001, *C. glabrata* ΔHT6, and *C. glabrata* Δ*mnn2* with and without antifungal agents. The quantification of the biomass is presented by abs/cm^2^. (* *p* < 0.05; *** *p* < 0.0005; **** *p* < 0.0001). Cg: *C. glabrata*; Flu: fluconazole; AmB: amphotericin B; Csf: caspofungin; Mcf: micafungin.

The effect of several drugs—Flu, AmB, Csf, and Mcf—was tested on *C. glabrata* ATCC2001, *C. glabrata* Δ*mnn2*, and *C. glabrata* ΔHT6 biofilms. The MBECs for *C. glabrata* ATCC2001 were determined previously [28,29,34] and used as the reference value for the other strains in this study. The results of the determination of the total biomass quantification are shown in Figure 2. In general, all strains showed a biomass reduction after contact with antifungals, but the response was not linear for the three *C. glabrata* strains. The controls revealed high capacities to produce biofilm, demonstrating that the lack of mannans in the mutant did not affect the quantity of biofilm produced. When the drugs were added, the mutant strain had a higher biomass loss, when compared with the controls and with *C. glabrata* ATCC2001 and *C. glabrata* ΔHT6 (Figure 2).

Table 1 shows the CFU count of the biofilm cells. When in contact with the drugs (MBEC), both the mutant and the parent strain were more tolerant to Flu, AmB, Csf, and Mcf, than ATCC2001.

Interestingly, only AmB was capable of reducing more than 1 Log_10_ of the initial CFU in the mutant strain, evidencing a higher resistance profile, when compared to the wild type strain, in which the echinocandins showed to have a good antifungal activity. Results demonstrate that, the initial CFU of the biofilm cells were very similar among the strains. It is, thus, probable that the differences in the antifungals susceptibilities were related to the biofilm matrices and/or in differences in the cell wall composition of each strain. In order to evaluate this, the composition of the cells walls and the biofilm matrices were also determined.

### 3.2. Effect of the Antifungals on Biofilm’s Cells Walls and Matrix

**Figure 3:**
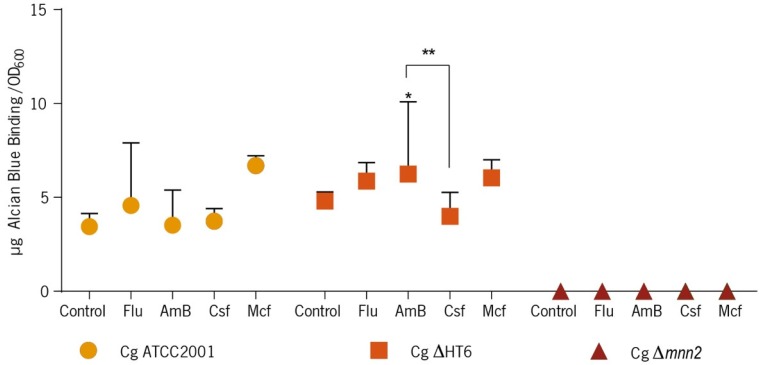
Alcian Blue binding assay. Data represent the mean amount of dye bound per biofilm cell of *C. glabrata* ATCC2001, *C. glabrata* ΔHT6, and *C. glabrata* Δ*mnn2* (undetected). (Cg—*C. glabrata*. * *p* < 0.05; ** *p* < 0.001).

So as to estimate the phosphomannans content in the biofilm cells, the quantitative Alcian Blue binding assay was performed (Figure 3).

The results showed that there was an increase in the mannans in the biofilm cells after the contact with antifungals drugs in *C. glabrata* ATCC2001 (Figure 3) and, comparing with the control group, *C. glabrata* ΔHT6 showed to have an increase in mannans only when Flu, AmB, and Mcf was applied to the biofilm, but not with Csf. Regarding *C. glabrata* Δ*mnn2*, the performance was different. The results showed that the KO of *MNN2* gene significantly reduced the ability of cells to bind to Alcian Blue, in comparison with the other strains, to a point that it could not be quantified (Figure 3).

**Figure 4:**
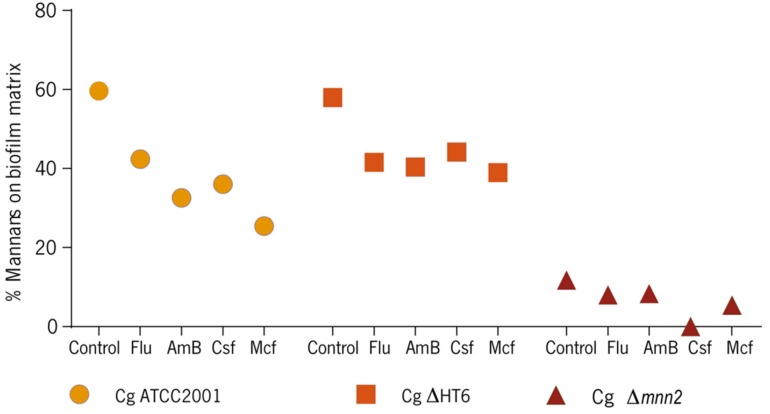
Alcian Blue binding assay in biofilm matrices of *C. glabrata* ATCC2001, *C. glabrata* ΔHT6, and *C. glabrata* Δ*mnn2*. Data represent the percentage of mannans on biofilm. (Cg—*C. glabrata*).

In addition, the content in mannans was also quantified on the biofilm matrices, which was, to authors’ knowledge, performed here for the first time (Figure 4).

In comparison with the controls, the results showed that all strains had less mannans content on the matrices in presence of antifungal drugs. Not surprisingly, the mutant had the lower quantity of these compounds on its matrices. In average, the controls had 50 to 60% of mannans in the matrices compositions, while *C. glabrata* Δ*mnn2* had less than 20% (Figure 4). The percentage decreased to about 20% after applying almost all drugs, for *C. glabrata* ATCC2001 and *C. glabrata* ΔHT6 and around 10% for the mutant, except for Csf, in which condition, no mannans were detected (*p* < 0.0005) (Figure 4).

**Figure 5:**
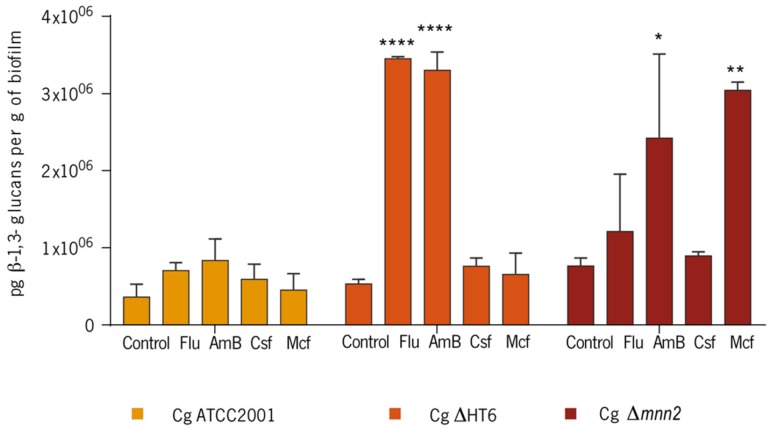
β-1,3 glucans concentration (pg/mL) on the biofilm matrices of *C. glabrata* ATCC2001, *C. glabrata* ΔHT6, and *C. glabrata* Δ*mnn2*. The values were normalised per dry weight of biofilm. (Cg—*C. glabrata*. * *p* < 0.05; ** *p* < 0.001; **** *p* < 0.0001).

The quantification of the β-1,3 glucans on the biofilm matrices of the three *C. glabrata* strains (Figure 5) showed that, comparing with the controls, the content of β-1,3 glucans increased in the matrices of *C. glabrata* ATCC2001. In the case of *C. glabrata* ΔHT6, particularly with Flu 1250 mg/L (*p* < 0.0001) and AmB 4 mg/L (*p* < 0.0001) there was a statistically significant rise in glucans production. Regarding *C. glabrata* Δ*mnn2* in contact with AmB 4 mg/L and Mcf 17 mg/L (*p* < 0.05 and *p* < 0.001, respectively), statistically significant rises were also detected. Curiously, although the mutant revealed to have less quantity of total polysaccharides in the biofilm matrix than the reference and the parent strain (data not shown), it showed to have the highest quantity of β-1,3 glucans per dry weight of biofilm matrix among all strains (7.66 × 10^5^ ± 1.03 × 10^5^), demonstrating a matrix richer in glucans’ polymers than the other two strains (Figure 5).

Afterwards, and in order to verify if the *C. glabrata* Δ*mnn2* initial cells were compensating the lack mannans of their cell walls with glucans, it was performed the quantification of β-1,3 glucans of the biofilm cells’ walls of the controls of the three strains (Table 2). The results confirmed that the content of β-1,3 glucans increased, in the mutant cell walls meaning that they have, indeed, a cell wall richer in β-1,3 glucans, when compared with the others (statistically significant).

## 4. Discussion

Systemic candidiasis is a worldwide emergent problem [1,35], associated with high mortality and high economic costs [33,34,35,36]. The fungal cell wall consists of β-1,3 glucans, β-1,4 glucans, β-1,6 glucans and chitin [10] and a layer of highly glycosylated mannoproteins [10,12,25]. Immunologically, these proteins called phosphomannans [16] are mainly organised in mannans, having a crucial role in adhesion and host recognition (host-fungus interactions) [9,13,37].

The results of the CLSM images (Figure 1) revealed that, microscopically among the strains, there were no variances on the cell wall, but the total biomass quantification (Figure 2) showed nonlinear biomass reduction after contact with antifungals. When compared with the other two strains, *C. glabrata* Δ*mnn2* demonstrated a higher biomass loss, which was assumed to be correlated to the KO in the *MNN2* gene and subsequently, the reduced quantity of mannans in its biofilm matrix (Figure 2).

The Alcian Blue is a cationic dye, binding to the negatively charged phosphate group of the phosphomannan [2,24,30]. This capacity is used to estimate the cell wall phosphomannan content [2,24,30]. The N-linked phosphomannan is attached to the branched mannan through α1,2-mannose residues, which extend out from the α1,6-mannose backbone. Consequently, a deletion of any the *MNN2* family members was expected to disturb the phosphomannan content of the cell wall [2]. *C. glabrata* ATCC2001 increased the quantities of mannans on the cell wall in the presence of drugs (Figure 3), which may be a possible adaptation of the cells to the stress caused by the antifungal agents. Similar cell walls adjustments during drug pressure have been described extensively as being directly responsible for antifungal resistance events [1,38,39,40]; compared with the control group, the parent strain (*C. glabrata* ΔHT6) increased the mannans content when stressed by Flu, AmB, and Mcf, but not by Csf (statistically significant, comparing with the AmB, *p <* 0.001), which demonstrated the intra-strain variability, genotypic and phenotypic alterations and also adaptable drug responses [41,42], but can also be a result of the molecular changes between Mcf and Csf. Both drugs have a cyclic peptide structure with an N-aryl group but with different patterns of hydroxylations and amino groups (R2 to R4). The N-aryl side chain (position R1) plays a critical role in the potency and toxicity and is the main point for chemical modification of the echinocandin analogues [43,44]. Csf is more hydroxylated and has more amino groups, while Mcf has more aryl groups. The substitution of the linoleoyl side chain with aryl side chains of low lipophilicity, nonlinear configuration or chains changed with highly polar groups end in loss of antifungal activity, proposing that planar, non-polar substitutions are critical for the antifungal activity [43,44]. Mcf molecule presents these modifications which are also a probable explanation for the differences found in this and in previous works of our group [29]. *C. glabrata* Δ*mnn2* considerably reduced the ability of cells to bind Alcian Blue (Figure 3), which has also been demonstrated on planktonic cells [9], suggesting that *MNN2* genes family are essential for phosphomannan integration, while deletion of other genes indicated a minor reduction in phosphomannan concentration [2,16]. This has been previously revealed for other species [14,16,45]. Those results raised the possibility that other polysaccharides’ metabolism (e.g., β-1,3 glucans) could be enhanced in the detriment of mannans in the mutant. Concerning the Alcian Blue binding assay in the biofilm matrices, all the *C. glabrata* strains showed to have less mannans on the matrices stressed by the presence of antifungal drugs, especially the mutant (Figure 4), which revealed that a poorer mannans’ matrix is more fragile, more sensible to environmental stresses and, thus, more susceptible to biomass loss (corroborating the biomass results).

Additionally, the β-1,3 glucans tended to increase their presence in the matrices when the biofilms were in contact with the drugs (Figure 5). It appears that, after a stress situation, the reduction in mannans, that was observed earlier (Figure 4), matches the β-1,3 glucans increase in *C. glabrata* (Figure 5). Moreover, the controls of the reference and the parent strains had ≈60% of mannans in the matrices compositions, while *C. glabrata* Δ*mnn2* had less than 20%. This reinforces the fact that the *C. glabrata* Δ*mnn2* biofilm matrix was mostly constituted by β-glucans, as it was determined (Figure 5). The quantification of β-1,3 glucans of the cells walls of the biofilm cells of the strains (Table 2) confirmed that the quantity of these biopolymers is variable in the cell walls of *C. glabrata* strains. The mutant displayed a cell wall richer in β-1,3 glucans, when compared with the others (statistically significant), which demonstrates higher capacities of compensation mechanisms, which, adding to the increase in the β-1,3 glucans content of the matrix, may be responsible for a more resistance profile to the drugs (Table 2).

The strong variability in the biofilm matrices and in the cell walls composition of *C. glabrata* has been formerly stated after any drug pressure, being related to higher pathogenicity and virulence states. Though, other factors are related to the biofilm drug resistance and one single feature is not able to clarify the complete phenomenon of resistance [23,34,46]. The KO of the *MNN2* gene has demonstrated to influence the drug response profile of biofilm cells, by inducing key changes in the matrices and cell wall’s compositions, validating again the great importance of β-glucans in the resistance of *C. glabrata* biofilms to antifungal drugs. Finally, this work also suggests that the identification and blocking of genes directly related to the plasticity of the composition of the matrices and cell walls are a good path to the search to new antifungal agents.

## Figures and Tables

**Table 1:** Log_10_ of CFU/cm^2^ ± Standard Deviation of biofilm cells using the minimum biofilm eradication concentrations (MBECs) of the reference strain (*C. glabrata* ATCC2001) for Flu, AmB, Csf, and Mcf for *C. glabrata* ΔHT6 and *C. glabrata* Δ*mnn2*.

Strain	Control	Flu	AmB	Csf	Mcf
*C. glabrata* ATCC2001	5.90 ± 0.18	4.95 ± 0.44	4.40 ± 0.30	3.80 ± 1.27	3.40 ± 0.07
*C. glabrata* ΔHT6	5.80 ± 0.36	3.69 ± 0.02	4.37 ± 0.45	5.42 ± 0.42	5.53 ± 0.41
*C. glabrata* Δ*mnn2*	6.16 ± 0.31	5.10 ± 0.43	4.23 ± 0.58	5.61 ± 0.11	4.98 ± 0.13

**Table 2:** β-1,3 glucans concentration (pg/mL) on the biofilm cells *C. glabrata* ATCC2001, *C. glabrata* HT6, and *C. glabrata* Δ*mnn2*. (* *p* < 0.05).

Strain	β-1,3 glucans Biofilm Cells Concentration (pg/mL) ± SD
*C. glabrata* ATCC2001	359.00 ± 8.20
*C. glabrata* ΔHT6	370.00 ± 5.08
*C. glabrata* Δ*mnn2*	387.00 ± 7.00 (*)

(SD—standard deviation).

## Supplementary Materials

The following are available online at http://www.mdpi.com/2218-273X/8/4/130/s1, Table S1: Fungal strains used in this study.
