# Supplementary material for: The MNN2 Gene Knockout Modulates the Antifungal Resistance of Biofilms of Candida glabrata
Source: Biomolecules. 2018 Oct 30;8(4):130. doi: 10.3390/biom8040130 (PMC6316230; doi:10.3390/biom8040130)
Supplement: Supplementary file 1 [file biomolecules-08-00130-s001.pdf]

biomolecules

MDPI

Supplementary material

Table S1. Fungal strains used in this study.

Species	Strain	Genotype or description	Source
C. glabrata	ATCC2001	Wild Type	ATCC
C. glabrata	ΔHT6	Δ*his3*::Sc*URA3* Δ*trp1*	[47]
C. glabrata	LJW-2P	Δ*his3*::*URA3* Δ*trp1* Δ*mnn2*::*HIS3* pCgACT14 (*TRP1*)	[9]
